# Epigenetic Marks at the Ribosomal DNA Promoter in Skeletal Muscle Are Negatively Associated With Degree of Impairment in Cerebral Palsy

**DOI:** 10.3389/fped.2020.00236

**Published:** 2020-06-03

**Authors:** Ferdinand von Walden, Rodrigo Fernandez-Gonzalo, Jessica Pingel, John McCarthy, Per Stål, Eva Pontén

**Affiliations:** ^1^Division of Pediatric Neurology/Orthopedics/Rheumatology, Department of Women's and Children's Health, Karolinska Institutet, Stockholm, Sweden; ^2^Department of Physiology, University of Kentucky, Lexington, KY, United States; ^3^Center for Muscle Biology, University of Kentucky, Lexington, KY, United States; ^4^Division of Clinical Physiology, Department of Laboratory Medicine, Karolinska Institutet, and Unit of Clinical Physiology, Karolinska University Hospital, Stockholm, Sweden; ^5^Department of Neuroscience, University of Copenhagen, Copenhagen, Denmark; ^6^Laboratory of Muscle Biology, Department of Integrative Medical Biology, Umeå University, Umeå, Sweden

**Keywords:** cerebral palsy, skeletal muscle, epigenetics, ribosome biogenesis, DNA methylation

## Abstract

**Introduction:** Cerebral palsy (CP) is the most common motor impairment in children. Skeletal muscles in individuals with CP are typically weak, thin, and stiff. Whether epigenetic changes at the ribosomal DNA (rDNA) promoter are involved in this dysregulation remains unknown.

**Methods:** Skeletal muscle samples were collected from 19 children with CP and 10 typically developed (TD) control children. Methylation of the rDNA promoter was analyzed using the Agena Epityper Mass array and gene expression by qRT-PCR.

**Results:** Biceps brachii muscle ribosome biogenesis was suppressed in CP as compared to TD. Average methylation of the rDNA promoter was not different between CP and TD but negatively correlated to elbow flexor contracture in the CP group.

**Discussions:** We observed a negative correlation between rDNA promoter methylation and degree of muscle contracture in the CP group. Children with CP with more severe motor impairment had less methylation of the rDNA promoter compared to less affected children. This finding suggests the importance of neural input and voluntary muscle movements for promoter methylation to occur in the biceps muscle.

## Introduction

Cerebral palsy (CP) is initiated by a non-progressive insult to the developing brain (1) and is the most common cause of motor impairment in children (2). Although the brain injury is stable in itself, motor symptoms typically worsen over time (3). Skeletal muscles of individuals with CP are weaker, thinner, and stiffer as compared to typically developed (TD) individuals (4, 5). Contracture formation, i.e., a progressive shortening of skeletal muscle, is believed to be a major contributing factor to poor function. The underlying cause of contracture formation is unknown, but believed to be multifactorial (6). The hypothesis of an early onset of impaired growth of the muscle has gotten increased attention during the last years, with several independent research groups presenting deficiencies in both skeletal muscle stem cell number (7) and function (8), and *de novo* synthesis of ribosomes in mature skeletal muscle of individuals with CP (9).

Ribosome biogenesis is crucial for skeletal muscle hypertrophy, and if impaired, skeletal muscle growth is stunted; for example, catch-up growth following early protein restriction is associated with attenuated ribosome biogenesis (10). We recently showed that adolescents with CP undergoing surgery due to fixed contractures of the elbow flexors display impaired skeletal muscle ribosome biogenesis as compared to age-matched typically developed adolescents (11). The rate-limiting step of ribosome biogenesis is believed to be transcription of the rDNA genes by RNA polymerase (Pol) I, producing the 45S pre-rRNA transcript. We observed that the 45S pre-rRNA transcript was less abundant in CP muscle vs. age-matched TD muscle, suggesting that skeletal muscle in CP has impaired growth potential (9).

The rDNA genes exist in several 100 copies throughout the genome, arranged in tandem repeats as clusters on 4–5 chromosomes, but not all genes are active at the same time. Out of these hundreds of gene copies, a subset is believed to be permanently deactivated by DNA methylation of the promoter region (12). Typically, tissues and cell types with high metabolic demand and a need for high synthetic capacity of protein have hypomethylated rDNA and thus higher rDNA transcriptional activity and higher abundance of the 45S pre-rRNA transcript. Conversely, in the event of a non-growth milieu such as malnutrition and/or reduced protein intake in mice, rDNA transcription is reduced and the rDNA promotor is typically methylated to a higher degree (13). While rDNA methylation may be a powerful regulator of ribosome biogenesis, essentially nothing is known about rDNA promoter regulation in human skeletal muscle in health or disease.

The purpose of the current investigation is to determine whether (1) rDNA promoter methylation in CP children differs from that of TD children, (2) decreased rDNA transcription observed in CP muscle is associated with hypermethylation of the promoter region, and (3) if rDNA promoter methylation is associated with severity of the motor impairment (i.e., Gross Motor Functional Classification System, GMFCS, and Manual Ability Classification System, MACS) and degree of contracture.

## Methods

### Participants

Nineteen children and adolescents (mean age, 15.5 years; range, 9–18 years; three females/16 males) with CP, scheduled for surgical lengthening of the biceps tendon or botulinum toxin injection of the biceps brachii, were included in the study. Their gross motor function, if and how well they could perform self-initiated movements especially regarding sitting, walking, and wheeled mobility, was classified on a five-level scale according to the GMFCS, Gross Motor Function Classification System. Individuals in GMFCS I had functional gross motor skills, while individuals in GMFCS V had severe limitations that impaired all voluntary movements. Individuals in GMFCS III need walkers and often use a wheelchair (14). Their ability to use their hands was classified on a five-grade scale according to MACS, the Manual Ability Classification System. The manual ability is classified irrespective of which hand is used or if both hands are used. It is thus also a measure of cognition as it affects the ability to use the hands if one or both have an impairment. Individuals in MACS I handle objects easily and successfully, and those in MACS III handle objects with difficulty and need help, while individuals in MACS V do not handle objects and require total assistance (15).

All patients had a developed contracture of the elbow flexor muscles, resulting in an extension deficit of the elbow. Passive range of motion of the elbow joint was measured with a goniometer. Full extension with a straight elbow was denoted as 0°, and any extension deficit (flexion contracture) was noted as x°.

Fifteen out of 19 samples were available for DNA analysis and 15 out of 19 samples for gene expression, although not all samples were from the same individuals. For subject details on sex, age, contracture, GMFCS, and MACS, see Table S1. Skeletal muscle samples were obtained intraoperatively under general anesthesia and were frozen in isopentane cooled on liquid nitrogen. All children had fasted a minimum of 10 h before surgery. Control skeletal muscle samples were obtained postmortem from children and young adults who had sustained accidental deaths (*n* = 10, mean age, 15.1 years; range, 7–21 years; two females/eight males, Table S2). All samples were stored in a −80°C freezer until analysis.

### RNA/DNA Extraction, cDNA Synthesis, and qPCR

Skeletal muscle biopsies (~25 mg) were homogenized in TRIzol reagent (Invitrogen, Carlsbad, CA) using a handheld homogenizer (Omni International, Kennesaw, GA). RNA and DNA were extracted according to the information provided by the manufacturer (Invitrogen). RNA was quantified using a NanoDrop VR 2000 (Thermo Scientific, Gothenburg, Sweden), and integrity assessed by agarose gel electrophoresis. DNA was resuspended in 8 mM NaOH, then centrifuged at 4°C at 12,000 × g to remove insoluble material. The DNA was transferred to a new tube and pH adjusted with HEPES: All samples were stored at −20°C until further use. Five hundred nanograms of RNA was reverse-transcribed with the VILO cDNA Synthesis Kit (Invitrogen) according to the manufacturer's recommendations. Fast SYBR Green Master Mix (Applied Biosystems™, Foster City, CA) was used for qPCR, in a QuantStudio 3 Real-Time PCR Systems machine (Thermo Fisher Scientific, Waltham, MA). PCR data were normalized by the geometric mean of three stable reference genes (*GAPDH, EMC7, VCP*). Primer sequences are available upon request. Melting curves were performed for every primer pair to confirm a single-product amplification. All samples were run in duplicates, and qRT-PCR data were analyzed using the −2ΔΔCT method.

### DNA Methylation Analysis

Quantitative methylation analysis was performed using the EpiTYPER methodology (16) and the MassARRAY® System (Agena Biosciences, San Diego) according to the manufacturer's recommendations and protocols. In the method, a targeted amplification of bisulfite converted DNA is followed by *in vitro* transcription, RNase cleavage, and subsequent fragment mass analysis by Matrix-Assisted Laser Desorption/Ionization Time of Flight Mass Spectrometry (MALDI-TOF MS) to quantify CpG sites.

PCR primers were adapted from D'Aquilla et al. (17). EpiTect-methylated and non-methylated bisulfite–treated control DNA (Qiagen) was used to evaluate the quantitative recapture of methylation ratios of the amplicons. The amplicon used in this study met the quality criteria of methylated and non-methylated data points measured at >79 and <5% methylation ratios, respectively, as well as standard deviation percentages <5%. Samples were run in duplicate, and standard deviation percentages >20% were removed from the study. The remaining data points correlated with *R*^2^ 0.72. Bisulfite conversion efficiency was evaluated by analyzing one non-CpG C:s in a small subset of the study samples. All data were checked by manually and visually inspecting the mass spectra.

### Statistical Analysis

Statistical analysis was performed with Prism 8 (GraphPad Software, Inc., CA). Values are reported as means ± *SD*. Differences in mRNA transcript levels and rDNA methylation percentage was assessed by the Student *t*-test (data normality investigated with Shapiro–Wilks test). Pearson's correlation coefficient (*r*) was used to investigate any potential correlation between rDNA promoter methylation, rDNA transcription, subject age, and elbow flexor contracture. Significance level was set at *p* < 0.05 for all statistical comparisons.

### Ethics

The ethical review board of Karolinska Institutet, Stockholm, Sweden, approved the study (Dnr 01-012, addendum 2018/1739-32, 04-324/2). The autopsy specimens were collected in agreement with Swedish laws and regulations on autopsy and transplantation with approval by the National Board of Health and Welfare. Informed written consent was obtained from all participants with CP and the parents of those younger than 18 years.

## Results

**Figure 1:**
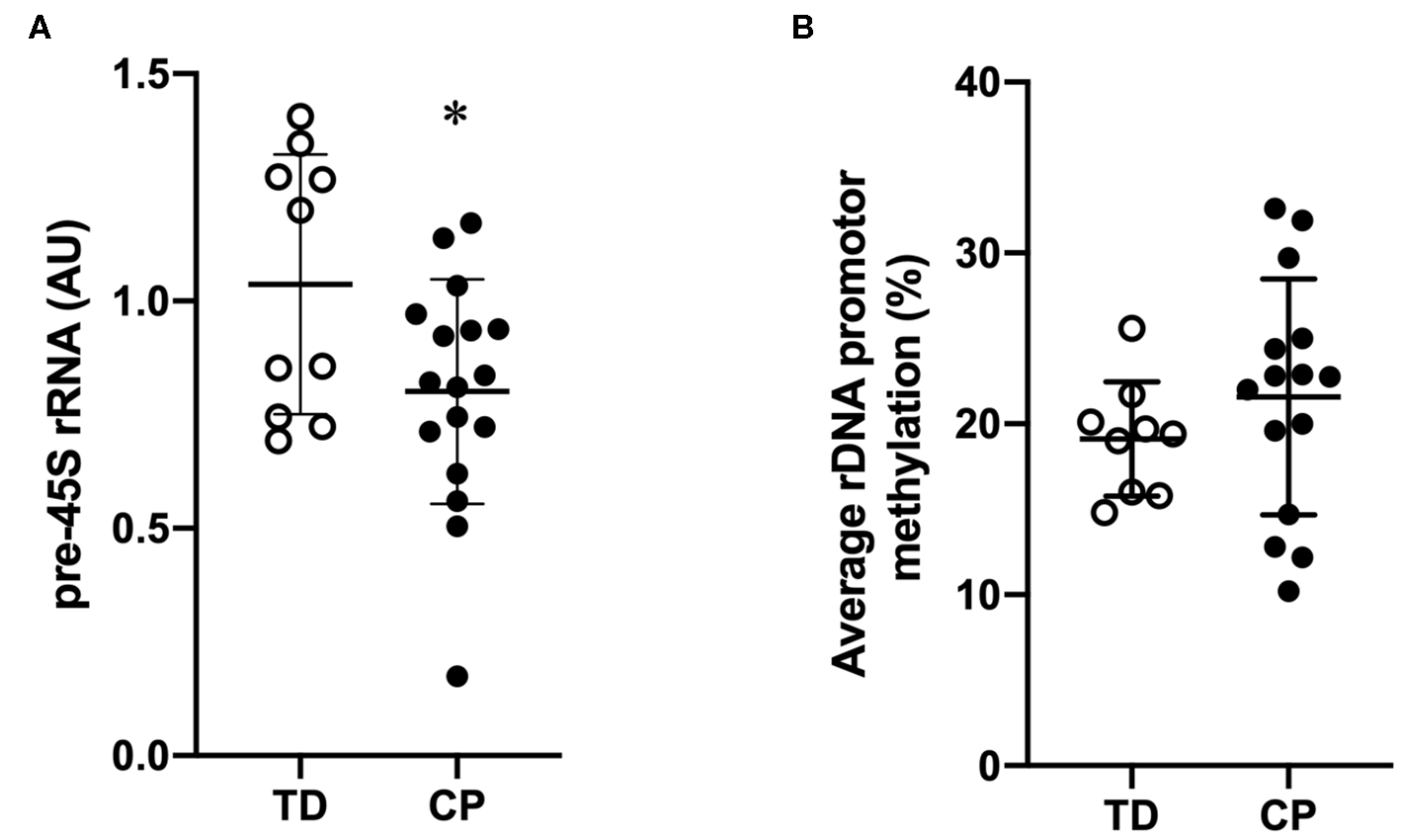
Suppressed transcriptional activity of rDNA genes but similar rDNA promoter methylation in skeletal muscle of children with cerebral palsy (CP) compared to typically developed (TD) children. **(A)** 45S pre-rRNA transcription (ITS-5.8S) in CP and TD (TD set as 100%). TD (open circles, *n* = 9) and CP (black circles, *n* = 15). Geometric mean of *GAPDH, EMC7*, and *VCP* was used for qRT-PCR normalization. **(B)** Average rDNA promoter methylation in CP and TD. TD (open circles, *n* = 9) and CP (black circles, *n* = 15). *denotes significantly different from TD, *p* < 0.05.

**Figure 2:**
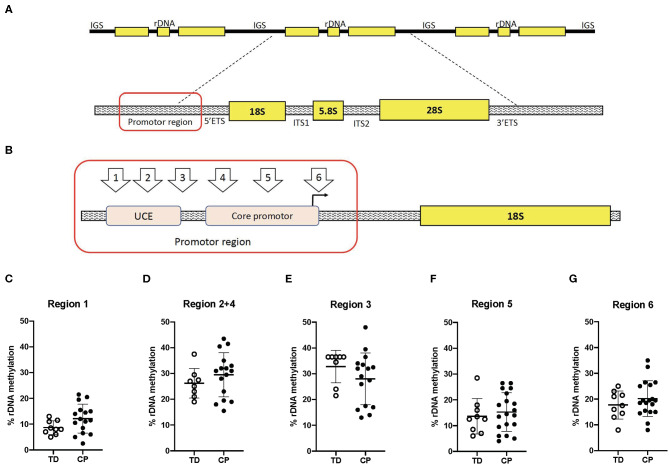
Ribosomal DNA methylation is not different at five unique sites in the Upstream control element (UCE) or the core promoter in skeletal muscle of children with cerebral palsy (CP) compared to typically developed (TD) children. **(A)** Schematic of the rDNA tandem repeats (top) and regions within one rDNA gene copy (bottom). IGS, Intergenic spacer; rDNA, ribosomal DNA; 5′ETS, 5′Externally transcribed spacer; ITS1, Internally transcribed spacer 1; ITS 2, Internally transcribed spacer 2, and 3′ETS, 3′Externally transcribed spacer. **(B)** Specification of investigated CpG sites within the rDNA promoter. UCE, Upstream core element. **(C–G)** Degree of methylation (%) of the rDNA promoter in CP and TD. TD (open circles, *n* = 8–9) and CP (black circles, *n* = 13–15).

**Figure 3:**
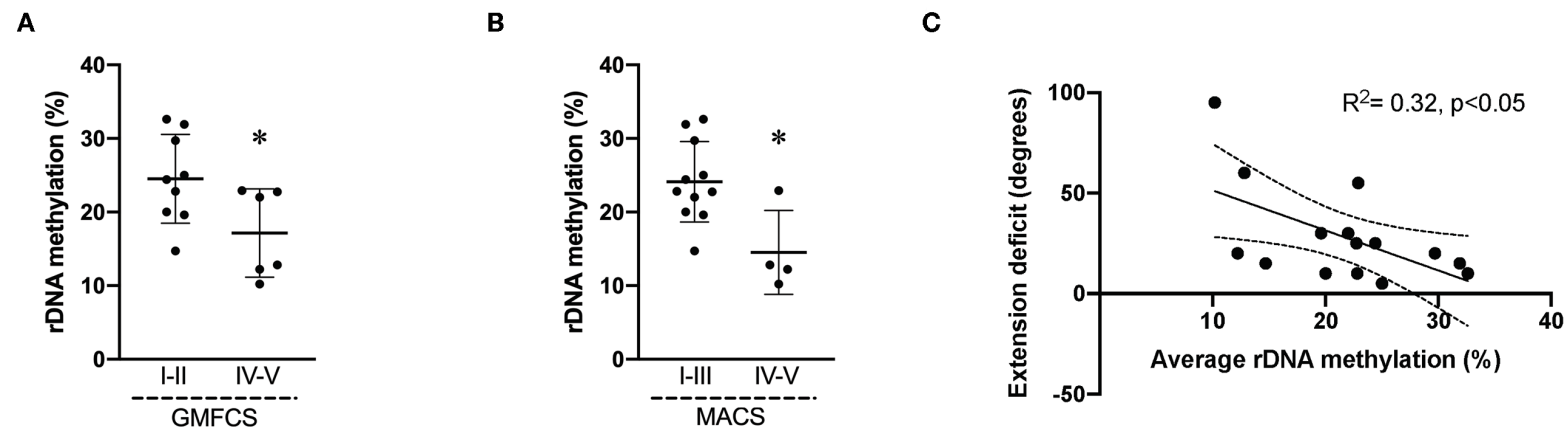
Average rDNA promoter methylation in cerebral palsy (CP) muscle negatively correlates to severity of impairment. Average promoter methylation (%) in children with CP divided accordingly to **(A)** GMFCS level I–II (*n* = 9) and GMFCS IV–V (*n* = 6); **(B)** MACS I–III (*n* = 11) and IV–V (*n* = 4). * denotes significantly different from GMFCS I–II and MACS I–III, *p* < 0.05. **(C)** Correlation analysis of average rDNA promoter methylation (%) and extension deficit (degrees) in CP children (*n* = 15). *R*^2^ and significance level indicated in graph.

Biceps muscle ribosome biogenesis, as indicated by 45S pre-rRNA abundance, was suppressed (−24%, *p* = 0.03 in CP as compared to TD (Figure 1A). The abundance of 45S pre-rRNA in the CP group was not related to age (*r* = −0.08, *p* = 0.77) or extension deficit of the elbow (*r* = −0.03, *p* = 0.92) and was not influenced by severity of CP. Average methylation of the rDNA promoter was not different between CP and TD (21.6 ± 6.9 [95% CI 18.1–25.1] vs. 19.1 ± 3.3 [95% CI 17.1–21.2], Figure 1B). In total, five unique CpG sites in the promoter were assessed (Figures 2A,B) and no statistically significant differences were seen between CP and TD (Figures 2C–G). Within the CP group, more severely affected children had a significantly lower percentage of methylated rDNA genes as compared to less affected children (GMFCS IV–V vs. GMFCS I–II; 24.5 ± 6.0 vs. 17.1 ± 6.0, *p* = 0.037 and MACS IV-V vs. MACS I-III; 24.1 ± 5.5 vs. 14.5 ± 5.7, *p* = 0.01). Moreover, the degree of methylation was inversely correlated to the flexion contracture of the elbow joint (*r* = −0.57, *p* = 0.03 in the CP group (Figures 3A–C), but not related to age (*r* = −0.14, *p* = 0.62) or 45S pre-rRNA levels (*r* = 0.30, *p* = 0.34) in either group.

## Discussion

This study addresses the effect of damage to the developing brain, i.e., CP on methylation of the rDNA promoter in skeletal muscle biopsies. We show that although rDNA methylation did not differ at the group level between CP and TD, the degree of promoter methylation within the CP group was related to severity of the disorder (GMFCS and MACS) and correlated negatively to flexion contracture of the elbow joint.

Reduced ribosome biogenesis has been linked to stunted catch-up growth following protein malnutrition in animal models (10) and muscle cells *in vitro* (11, 18) and has been suggested to, at least in part, explain anabolic resistance in the elderly (19). We have previously shown that ribosome biogenesis is reduced in CP muscle and that this is associated with reduced protein levels of key transcription factors, e.g., UBF and TIF-1A, regulating RNA polymerase (Pol) I activity (11). In this report, we aimed to provide further insight into the reduced *de novo* synthesis of rRNA in CP muscle by exploring CpG methylation of the rDNA promoter. Increased degree of methylation within the promoter region of a protein coding gene is typically repressive of gene activity whereas reduced methylation of the promoter region has a stimulatory effect on gene expression (20, 21). Likewise, promoter methylation of the rDNA genes has been shown to influence RNA Pol I activity and 45S pre-rRNA abundance in different cancers, however in a less clear manner. Methylation at the rDNA promoter correlated negatively with 45S pre-rRNA abundance in hepatocellular carcinoma (22) and CD34+ cells in patients with myelodysplastic syndrome (23). Other studies have shown the opposite or no relationship between methylation of the rDNA promoter and rDNA transcriptional activity (24, 25). In our study, 45S pre-rRNA levels did not correlate to rDNA promotor methylation in neither TD nor CP muscle; this is the first information on rDNA promoter methylation in human skeletal muscle. Moreover, 45S pre-rRNA levels did not correlate to contracture severity or GMFCS level in individuals with CP. Thus, the effect of rDNA promoter methylation on Pol I transcription in skeletal muscle is not fully understood and tentatively suggests other modes of regulation. The varying number of rDNA genes from individual to individual likely influences transcription and results in a less clear regulatory role of promotor methylation for gene output, as at least in theory, an individual with a higher copy number of rDNA genes could still maintain a high level of transcription despite a high average promotor methylation. As we did not assess rDNA copy number in this study, a more detailed investigation of rDNA gene regulation in CP muscle is beyond the scope of the current report.

In the current study, we observed that children categorized as having a more severe impairment, i.e., in the more severe GMFCS and MACS categories, had a lower percentage of methylation of the promoter region, together with a negative correlation between muscle contracture of the biceps brachii and percentage of methylation of the promoter region in CP muscle. In line with these data, the severity of manual ability (MACS) and contractures in CP are known to correlate (26). A tentative interpretation of our results could be that typical neuromuscular development and motor activity shape the methylation landscape of the rDNA promoter and that a brain insult at an early age offsets this epigenetic imprinting. Thus, voluntary muscle use could be a factor of importance for establishing the methylome of the rDNA genes in skeletal muscle. There is to our knowledge currently no information about the effects of physical activity, neither acute nor chronic, on CpG methylation of the rDNA genes in skeletal muscle. Future studies are warranted to determine the effect of rDNA methylation on rDNA transcription and muscle size regulation in health and disease.

A more speculative explanation is that more-affected children with CP have been suggested to have an inadequate one-carbon metabolism and thereby DNA methylation capacity (27). Data supporting this claim was published by Schoendorfer et al. showing altered red blood cell volume and deranged biochemical markers of the methylation cycle in children with CP as compared to TD control children (27). The authors suggest that the underlying cause for the reduce methylation capacity could be due to poor nutrition, a well-known phenomenon, as almost 45–50% of children with CP have been reported to be undernourished (28, 29). However, speaking against a general inability to methylate DNA, as suggested by Schoendorfer et al. is a series of papers published in recent years that investigate differences in global DNA methylation between CP and TD children, i.e., DNA methylation in white blood cells. Two of these studies are case (CP) vs. control (TD) studies (30, 31), and two are studies on monozygotic twins discordant for CP (32, 33). All of these studies indicate that distinct epigenetic imprinting is evident in CP and that this is detectable very early on, even before 1 year of age. A recent study has observed a positive effect of a demethylating agent, i.e., 5-azacytidine (5-AZA), on the functionality of isolated skeletal muscle stem cells (satellite cells) from individuals with CP in culture (8). However, the differentially methylated genes in CP include both hypermethylated and hypomethylated genes as compared to TD children, clearly showing the dynamic nature of the DNA methylome in CP. Thus, our results and previously published data suggest that a treatment such as 5-AZA targeting a single epigenetic event (DNA methylation) is likely not a successful therapy on a large scale in a clinical setting. For example, in one of the aforementioned studies, among the top 200 differentially methylated genes in white blood cells in CP, roughly half were hypomethylated as compared to TD children (30). With this in mind, a clear and noteworthy difference between the aforementioned studies and our investigation is the level of specificity, related to both the target gene (rDNA) and the target organ (skeletal muscle rather than global methylation of nucleated blood cells).

The results of the current investigation should be viewed in light of its limitations. The sample size is low, which limits the statistical power of the study. Thus, although we did not observe any difference in rDNA promoter methylation between TD and CP subjects, a better-powered study is needed to be able to confirm these results. All skeletal muscle biopsies from adolescents with CP were taken from skeletal muscle with a fixed contracture; thus, only correlations could be made and no causation assessed. Moreover, as all muscle samples from TD subjects were collected post-mortem following an accidental death, no information on pROM of the elbow joint is available for TD subjects.

In conclusion, the reduced ribosome biogenesis in CP as compared to TD individuals was not related to rDNA promoter methylation. Interestingly, children classified as having a more severe CP had lower percent rDNA promotor methylation. Moreover, we observed a negative correlation between rDNA promoter methylation and flexion contracture. This finding suggests the importance of neural input and voluntary muscle movements, significant for people in the less severe GMFCS and MACS groups, for rDNA promoter methylation to occur. More research is needed on epigenetic changes in CP skeletal muscle and the potential influence of such modifications on growth and contracture development.

## Data Availability Statement

The datasets generated for this study are available on request to the corresponding author.

## Ethics Statement

The studies involving human participants were reviewed and approved by the ethical review board of Karolinska Institutet, Stockholm, Sweden (Dnr 01-012, addendum 2018/1739-32, 04-324/2). Written informed consent to participate in this study was provided by the participants' legal guardian/next of kin.

## Author Contributions

FW designed study, performed experiments, analyzed data, interpreted results, designed figures/tables, drafted manuscript, edited manuscript, and approved final version of manuscript. RF-G and PS performed experiments, edited manuscript, and approved final version of manuscript. JP and JM interpreted results, edited manuscript, and approved final version of manuscript. EP designed study, interpreted results, edited manuscript, and approved final version of manuscript.

## Conflict of Interest

The authors declare that the research was conducted in the absence of any commercial or financial relationships that could be construed as a potential conflict of interest.
