# Supplementary material for: Epigenetic Marks at the Ribosomal DNA Promoter in Skeletal Muscle Are Negatively Associated With Degree of Impairment in Cerebral Palsy
Source: Front Pediatr. 2020 Jun 3;8:236. doi: 10.3389/fped.2020.00236 (PMC7283884; doi:10.3389/fped.2020.00236)
Supplement: Supplementary file 1 [file Table_1.DOCX]

Table 1 . Cerebral Palsy subject details

| **Subject number** | **Sex** | **Age at surgery** | **GMFCS** | **MACS** | **Ext. def.** | **qRT-PCR** | **Agena Epi-Typer** |
| --- | --- | --- | --- | --- | --- | --- | --- |
| CP #1 | Male | 15 | I | II | 15 |  | x |
| CP #2 | Male | 16 | I | I | 5 |  | x |
| CP #3 | Male | 17 | II | II | 15 | x | x |
| CP #4 | Male | 17 | IV | III | 30 | x | x |
| CP #5 | Male | 16 | I | II | 10 | x | x |
| CP #6 | Male | 9 | I | II | 10 |  | x |
| CP #7 | Male | 13 | IV | III | 25 | x | x |
| CP #8 | Male | 17 | V | V | 60 | x | x |
| CP #9 | Female | 17 | V | V | 20 |  | x |
| CP #10 | Female | 18 | V | V | 55 | x | x |
| CP #11 | Male | 17 | I | II | 30 | x | x |
| CP #12 | Female | 16 | V | V | 95 | x | x |
| CP #13 | Male | 17 | II | II | 25 | x | x |
| CP #14 | Male | 9 | I | I | 15 | x |  |
| CP #15 | Male | 15 | I | III | 20 | x | x |
| CP #16 | Male | 17 | I | II | 10 | x | x |
| CP #17 | Male | 15 | I | I | 5 |  |  |
| CP #18 | Male | 16 | IV | III | 0 | x |  |
| CP #19 | Male | 15 | IV | IV | 20 | x |  |

CP – Cerebral Palsy, GMFCS – Gross Motor Function Classification Scale, MACS - Manual Ability Classification System, Ext. def. – Extension deficit at the elbow joint in degrees, qRT-PCR –Quantitative Reverse Transcription Polymerase Chain Reaction
