# Supplementary material for: Epigenetic Marks at the Ribosomal DNA Promoter in Skeletal Muscle Are Negatively Associated With Degree of Impairment in Cerebral Palsy
Source: Front Pediatr. 2020 Jun 3;8:236. doi: 10.3389/fped.2020.00236 (PMC7283884; doi:10.3389/fped.2020.00236)
Supplement: Supplementary file 2 [file Table_2.DOCX]

Table 2. Typically developed control subjects

| **Subject number** | **Sex** | **Age at biopsy** | **qRT-PCR** | **Agena Epi-Typer** |
| --- | --- | --- | --- | --- |
| TD #1 | F | 7 | x | x |
| TD #2 | F | 13 | x | x |
| TD #3 | M | 11 | x | x |
| TD #4 | M | 14 | x |  |
| TD #5 | M | 18 | x | x |
| TD #6 | M | 16 | x | x |
| TD #7 | M | 21 | x | x |
| TD #8 | M | 18 | x | x |
| TD #9 | M | 19 | x | x |
| TD #10 | M | 14 | x | x |

qRT-PCR – Quantitative Reverse Transcription Polymerase Chain Reaction
